# An Uncommon Reaction to a Very Common Drug: Hydrochlorothiazide-Induced Pulmonary Edema

**DOI:** 10.7759/cureus.100353

**Published:** 2025-12-29

**Authors:** Montana S Gunter, Vanessa Velazco, Thomas Neal, Karl Kuhn, Tanner Boyd

**Affiliations:** 1 Pharmacy, Williamson Medical Center, Franklin, USA; 2 Pulmonology, Williamson Medical Center, Franklin, USA

**Keywords:** adverse reaction, hydrochlorothiazide, non-cardiogenic pulmonary edema, pulmonary edema, thiazide diuretic

## Abstract

Non-cardiogenic pulmonary edema is a rare but serious adverse reaction to hydrochlorothiazide. We present a case report of a patient who developed severe dyspnea and hypoxemic respiratory failure within 30 minutes of the first hydrochlorothiazide dose. Management consisted of supportive care, oxygen therapy, and avoidance of thiazide diuretics, consistent with prior reports. This case emphasizes the importance of recognizing hydrochlorothiazide-induced non-cardiogenic pulmonary edema as a rare but life-threatening adverse event, the limitations of relying solely on biomarkers such as procalcitonin, and the need for careful documentation to prevent re-exposure.

## Introduction

Hydrochlorothiazide (HCTZ) is a commonly prescribed thiazide diuretic with a favorable safety profile. Common adverse effects include hypokalemia, hypomagnesemia, orthostatic hypotension, and hyperuricemia. However, non-cardiogenic pulmonary edema (NCPE) is a rare but serious side effect that has been reported previously in the literature [1]. The mechanism of this reaction is largely unknown, but proposed mechanisms include an immunologically mediated capillary leak syndrome, possibly triggered by a hypersensitivity reaction [1-3]. Some cases demonstrate decreased serum IgG and increased IgM and complement levels, suggesting immune activation, but specific IgE or T-cell-mediated mechanisms have not been consistently demonstrated [4,5]. This case report will describe a patient who presented with acute hypoxic respiratory failure after ingestion of HCTZ.

## Case presentation

**Figure 1:**
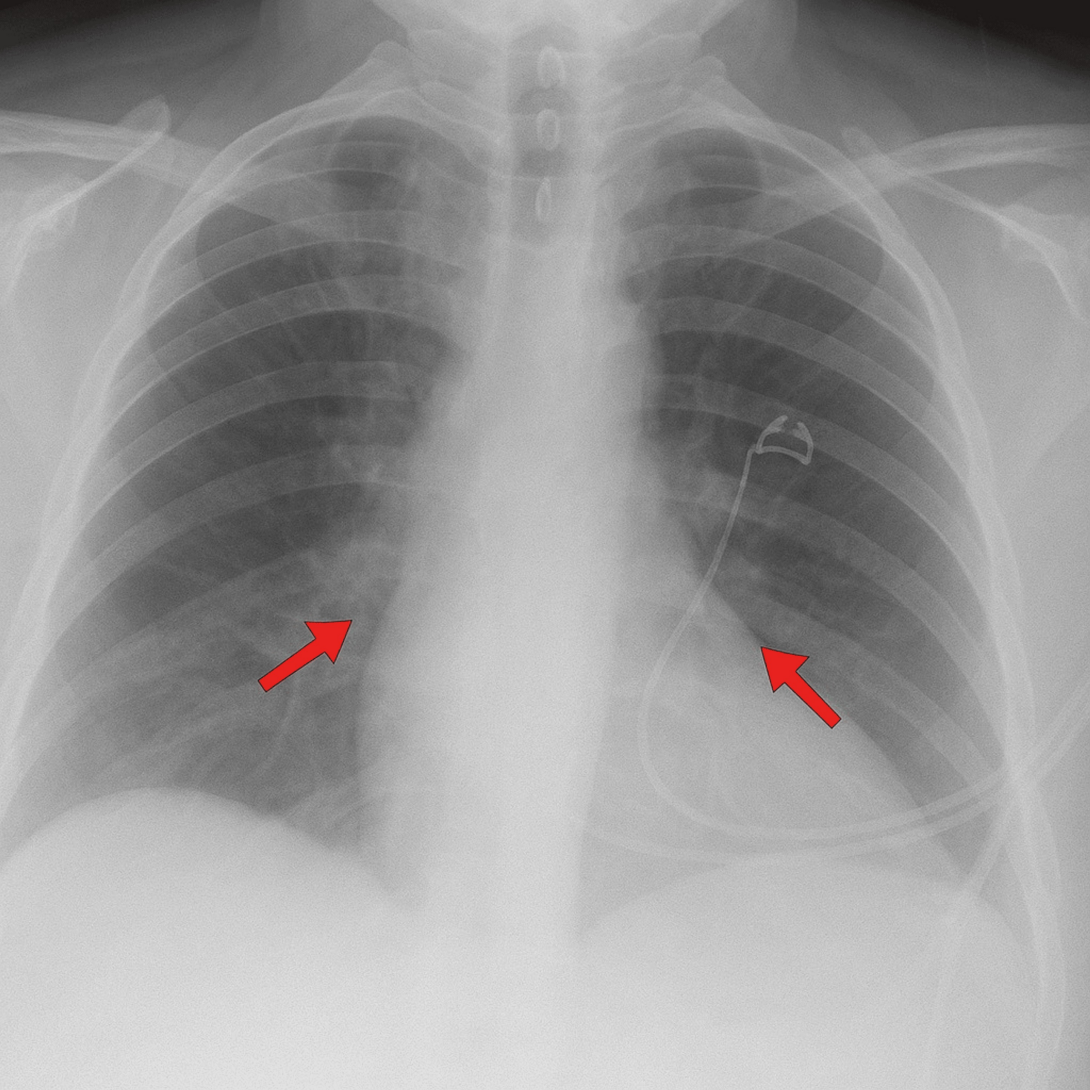
Chest X-ray demonstrating bilateral pulmonary opacities

A 50-year-old female presented to the emergency department with complaints of shortness of breath, nausea, and vomiting. She denied any fever, chills, abdominal pain, or diarrhea. Her past medical history includes hypertension and osteoarthritis for which she was taking lisinopril, HCTZ, and celecoxib. Most recently, her lisinopril dose was increased from 20mg to 40mg and she was newly initiated on HCTZ at 25mg. The patient reported that symptoms began 30 minutes after taking her first dose of HCTZ. Her vitals upon presentation included a blood pressure of 180/120 mmHg, a pulse of 138 beats per minute, a respiratory rate of 36 breaths per minute, and a temperature of 97.7 °F. A repeat blood pressure of 133/80 was taken an hour later. A low-grade fever of 100.4 °F was reported an hour after presentation but resolved in approximately two hours. Physical exam was unremarkable with the exception of diffuse rales heard throughout all lung fields. Chest X-ray showed bilateral pulmonary opacities (Figure 1). Bilateral lower lobe and right middle lobe pulmonary opacities were consistent with pulmonary edema. No large pleural effusion was noted and there was no focal infiltrate suggestive of pneumonia. Echocardiogram was unremarkable. Notable labs include a white blood cell count (WBC) of 1.42 x 103/mm3, hemoglobin of 17.1 g/dL, and procalcitonin of 10.3 ng/mL (Table 1). Initial troponin peaked at 0.130 ng/ml and decreased to 0.054 ng/ml within 24 hours. Empiric antibiotics were started for community-acquired pneumonia coverage and the patient was placed on bilevel positive airway pressure (BiPAP) to improve oxygenation. She was given a single dose of ethacrynic acid 50mg for pulmonary edema. BiPAP was taken off that evening and she was placed on a nasal cannula. The following morning, the patient's leukopenia resolved, with a WBC count of 11.18 x 103/mm3 and improvements in oxygenation demonstrated by removal of nasal cannula and a walking desaturation study on room air. Her vitals were all within normal limits. HCTZ was added to her allergy list and the patient was discharged after one day to complete antimicrobial therapy and a follow-up appointment with a primary care provider.

**Table 1:** Baseline laboratory results

Parameter	Patient Value	Reference Range	Units
Hemoglobin	17.1	11.5-15.5	g/dL
Hematocrit	53.2	34-45	%
Platelet	273	150-400	/L
White Blood Cells	1.42	4,000–10,700	/L
Procalcitonin	10.3	<0.077	ng/mL
Troponin	0.13	0.000-0.034	ng/mL

## Discussion

This patient's case is consistent with other reports, with severe dyspnea beginning within 30 minutes of the first HCTZ dose, hypoxemic respiratory failure with bilateral opacities on chest X-ray, rapid clinical improvement after discontinuation of the drug, and absence of an infectious etiology [6-8]. Although the exact pathophysiology remains unknown, it is clear on exam that there is fluid buildup in the lungs. Therefore, ethacrynic acid was given to provide diuresis to the patient. This was chosen as it is a loop diuretic without a sulfa side chain. A reaction to the sulfonamide group on HCTZ was later ruled unlikely as the patient was taking celecoxib, a non-steroidal anti-inflammatory drug with a sulfonamide group in its chemical structure.

A diagnostic challenge in this case was the elevated procalcitonin and the empiric initiation of antibiotics. Although procalcitonin is most often used as a biomarker for bacterial infection, the literature documents that procalcitonin can be elevated in noninfectious systemic inflammatory states and severe drug reactions. Therefore, a high level should not automatically be taken as proof of bacterial pneumonia. The rapid clinical recovery on room air by the following day, resolution of leukopenia, and the short interval between drug ingestion and symptom onset favor a drug-induced NCPE over primary community-acquired pneumonia in this patient.

Management primarily consists of supportive care such as oxygenation support, immediate discontinuation of the drug, and careful observation. Given the potential severity, as some case reports have reported use of extracorporeal membrane oxygenation, it is pertinent to educate patients on the likelihood of reoccurrence and to refrain from taking a thiazide diuretic [9]. Clinicians should also document the reaction in the medical record and add HCTZ to the allergy/adverse reaction list, as was done for this patient.

## Conclusions

This case highlights a rare but serious adverse reaction to hydrochlorothiazide, presenting as non-cardiogenic pulmonary edema with rapid onset and resolution after drug discontinuation. Recognition of this reaction is critical, as it can mimic infections such as pneumonia or sepsis, and biomarkers such as procalcitonin may be misleading in this context. Clinicians should ensure prompt discontinuation, provide supportive care, and document the reaction to prevent future thiazide exposure.
